# Post-Transplant Complications in Patients Undergoing Autologous Hematopoietic Cell Transplantation (HCT)—A Comparative Analysis of Home Care versus Hospitalized Patients

**DOI:** 10.3390/medicina60010044

**Published:** 2023-12-26

**Authors:** Ana María Garcés-Carrasco, Enric Santacatalina-Roig, Carlos Carretero-Márquez, Elena Chover-Sierra, Antonio Martínez-Sabater, Evelin Balaguer-López

**Affiliations:** 1 Oncology and Hematology Department, Hospital Clínico Universitario de Valencia, 46010 Valencia, Spainenric.santacatalina@uv.es (E.S.-R.);; 2 Nursing Department, Facultat d’Infermeria i Podologia, Universitat de València, 46010 Valencia, Spain; elena.chover@uv.es (E.C.-S.); evelin.balaguer@uv.es (E.B.-L.); 3 Nursing Care and Education Research Group (GRIECE), GIUV2019-456, Nursing Department, Universitat de Valencia, 46010 Valencia, Spain; 4 Internal Medicine Department, Consorcio Hospital General Universitario de Valencia, 46014 Valencia, Spain; 5 Grupo Asociado de Investigación en Cuidados (INCLIVA, Hospital Clínico Universitario de Valencia, 46010 Valencia, Spain

**Keywords:** transplantation, autologous, home nursing, hematology, hematopoietic cell transplantation, adverse effects

## Abstract

*Background and Objectives*: The increase in indications for hematopoietic cell transplants (HCTs) has led to the development of new care options after said transplant, such as home care after transplantation, which improves the patients’ quality of life. The main purpose of this research is to analyze the differences in the appearance of post-transplant complications between patients having underwent autologous HCT with at-home post-transplant modalities and those under in-hospital post-transplant care. *Materials and Methods*: An observational, analytical, longitudinal, and retrospective study of cases and controls. All transplanted people in the domiciliary model since 2020 are included as cases (20 subjects). For each case, two controls (40 subjects) are proposed among patients who received an autologous transplant in a hospital in the last five years with a similar demographic and pathological base profile in each case. *Results*: No significant differences were found between cases and controls, except for the Karnofsky value, which was higher in people receiving home treatment (91.7% vs. 87.74%; *p* = 0.05). The average number of days of the process post-transplantation was more significant at home (processing days 22.4 ± 2.6; post-transplantation days of 16.4 ± 2.08 versus 21.21 ± 4.18, with a mean of 15.51 ± 3.96 days post-transplant (days of the process *p* = 0.022; days post-transplant *p* = 0.002)). There is a more significant presence of neutropenic fever, mucositis, and positive blood cultures in the post-transplant patients who remain in the hospital. In contrast, the patients receiving home care post-transplantation undergo significantly more weight loss. Regarding the odds ratio of the appearance of adverse events, in the hospital setting, it is up to 8.5 times more likely to encounter neutropenic fever, 4.63 times more likely for mucositis, and 6.65 times more likely for the presence of pathogens in blood cultures. *Conclusions*: The home care modality in the post-transplant phase does not show an inferiority in conditions in the management and safety of the patient concerning the appearance of adverse events. However, more significant weight loss is detected in patients at home, and an increased risk of episodes of neutropenic fever, mucositis, and positive blood cultures for patients in hospital settings.

## 1. Introduction

In recent decades, the improvement in the efficacy and efficiency of hematopoietic cell transplantation (HCT) has led to an increase in previous indications [1,2], with benefits appearing in other processes [2,3] and leading to treatments with better survival rates or better remission durations [4]. In 2020, it was estimated that 544,352 non-Hodgkin lymphomas (NHLs), 474,519 leukemias, 176,404 multiple (MM), and 83,087 Hodgkin lymphomas (HLs) were diagnosed worldwide [5]. Approximately 50,000 people undergo yearly bone marrow transplants (BMTs) [6]. In our country, 3375 HCTs were performed in 2020, of which 78 were performed in our hospital [5]. HCT involves the infusion of hematopoietic progenitor cells into a patient, sourced from potential reservoirs such as bone marrow, peripheral blood, or umbilical cord blood. These cells may be obtained from different types of donors, including autologous (from the patient themselves), syngeneic (from an identical twin), or allogeneic (from a genetically distinct donor). HCT is employed when the regenerative capacity of hematopoietic tissue is insufficient due to a primary disease of the bone marrow (BM) or to having received intensive chemo or radiotherapy treatment [6]. It is a procedure indicated in many malignant and non-malignant, congenital, and acquired hematological diseases, which is used as first-line treatment or when conventional medicine fails (Hodgkin’s lymphomas, multiple myelomas, marrow aplasias, and others) [7].

After the indication of the process, it begins with a conditioning phase that consists of the administration of doses of chemotherapy or radiotherapy to destroy the tumor cells (myeloablative, non-myeloablative, or of reduced intensity) and allow for the grafting effect to be facilitated, avoiding the rejection of the newly transplanted cells [8]. After this phase, the hematopoietic progenitors are infused (the day of infusion is called day 0). Altogether, the entire HCT process has an average duration of 21 days (since conditioning recovery to final phase). This process can cause side effects (nausea or vomiting, diarrhea, oral mucositis, alopecia, mumps, neutropenia infections, bleeding, anemia, hemorrhagic cystitis, implant syndrome, and graft-versus-host disease (GVHD)) and affect the quality of the patient’s life [9,10,11,12,13,14].

HCT is physically and psychologically challenging, potentially exposing patients to quality of life (QoL) deterioration [15]. The results reported by the patient should be evaluated [16], and they should cover the different dimensions of the quality of life [16]. This assessment should be extended to caregivers and family members due to the impact of the process and hospitalization on family health [17,18,19]. The presence of clinical complications increases the effect of the transplant on the patient and families, so it is essential to detect the affectation through the assessment of people which allows for adequate clinical decision-making [9,20] and to provide specific interventions to increase care [9,21,22]. Thus, the techniques for adapting the treatments and the different support measures for individuals and families receiving HCT are essential to improve the results of the QoL [15]. Educational interventions to enhance knowledge about side effects, risks, complications, and preventive behavior can reduce psychological distress and improve quality of life (QoL) [10,23]. 

One aspect that affects the patients’ quality of life is the lengthy hospitalization process that implies a change in the living conditions of the person and family and modifications to their routines and habitual patterns of behavior [24,25,26,27,28,29]. On the other hand, there has been a trend in recent years, not only for economic reasons but also for the effectiveness of the treatments, to transfer complex medical treatments to the home when possible, which triggered different initiatives to develop home transplants worldwide [30,31]. These studies assess other aspects, such as complications, drug use in hospitals and at home, and the impact of distance to the health centers [18,24,32,33,34]. In our country, a pilot experience of home autologous hematopoietic cell transplantation (HCT) started in 2015 [34], following the lines of similar programs developed in Europe [35]. The results of this project indicated the presence of shorter periods of febrile neutropenia, fewer episodes of fever, and a lower rate of readmissions, as well as a reduction in costs and hospital care pressure [25]. 

After the start of the COVID-19 pandemic in 2020 [36], it was necessary to reorganize the care activity, promoting the implementation of a home hospitalization program for autologous HCT in our center. This program considered hospitalization for the conditioning and infusion phases and home transfer for the third transplant or post-transplant and recovery phases. In its first year of implementation, 20 autologous HCTs were performed on an outpatient basis in patients diagnosed with MM and lymphoma. 

In this program, those patients without severe comorbidities (nephropathies, heart diseases, etc.), with homes close to the hospital (<30 km), and with adequate family support were included. According to the center’s protocol, the patient is admitted to the hospital for preparation, including PICC insertion, and is transferred home the day after the reinfusion of hematopoietic cells for the post-transplant phase. At home, follow-up is carried out every 12 h by nursing (by telephone or in-person), including control of vital signs and administration of treatment until the end of the process. Given the possibility of worsening, at all times, there is a bed reserved in the hospital for home transplant patients and constant telephone contact with nurses and hematologists. There are few home transplant units in Spain and little bibliography on the improvements this care model entails compared to the traditional hospital model. Hence, the primary objective of this study is to examine disparities in the occurrence of post-transplant complications between patients undergoing this treatment in the home transplant modality and those followed in the hospital transplant modality.

## 2. Materials and Methods

A retrospective observational study analyzed matched cases and controls in the home hematopoietic cell transplantation (HCT) care model. The study spanned from 20 September 2020 to 30 November 2021, with a sample of adult patients. Each case from the home HCT group had two controls matched for diagnosis, comorbidities, disease stage, sex, and age [37,38]. We aimed for 20 cases and 40 controls to achieve a minimum odds ratio of 6.62, assuming a control group exposure rate of 0.42 [39].

Both groups underwent comprehensive variable collection to outline pre-transplant baseline characteristics. Simultaneously, post-transplant adverse effects data, including febrile episodes, days of neutropenia, gastrointestinal complications, and infections, were gathered from infusion day to the process’s conclusion. Retrieval involved reviewing each patient’s clinical computerized records in hospital databases. Case and control selection and about three weeks of data collection by a second investigator preceded audit oversight by a third investigator before analysis. Given the study’s retrospective nature, potential measurement and selection biases were acknowledged [38]. An initial training session clarified variable definitions and located patient records to address observation bias. A second observer independently reviewed 5% of cases, with included controls ensuring proper registration.

Initially, data collection from patients included in the home care model was carried out. Subsequently, based on the baseline conditions of these patients, controls were recruited retrospectively and consecutively for each case. Once the controls were detected, data collection and tabulation were carried out using the same methodology for household cases.

Descriptive analysis by groups of the pre-transplant baseline conditions, conditioning phase, transplant phase, and post-transplant treatment was carried out, as well as a description of post-transplant adverse effects. 

When parametric criteria were met to identify significant differences, the t-student statistic was applied to quantitative variables and the Chi-squared statistic to qualitative variables. The Mann–Whitney U statistic for quantitative variables and Fisher’s exact F statistic for qualitative variables were chosen for non-parametric criteria. In case of significant differences in adverse effects, the risk ratio (odds ratio) was calculated using logistic regression. All analyses maintained a confidence level of 95% with an alpha of 0.05%. Statistical analysis was performed using SPSS 21 (IBM).

This study was conducted following the Declaration of Helsinki and approved by the Ethics Committee of the University Clinical Hospital of Valencia (protocol code 2022/27 (377)) at 27 January 2022.

## 3. Results

A sample of 20 patients whose post-transplantation was carried out at home and 40 patients whose post-transplantation setting was a hospital, specifically in the transplant area of the Hematology Unit, was analyzed. No significant differences were found in the baseline conditions of the patients, except for the Karnofsky scale (*p* = 0.050), whose score was higher in patients at home (91.7%) than in patients in the hospital (87.74%) (Table 1).

Regarding the conditioning phase compared to the home or hospital post-transplant context, it must be said that both groups are very similar. In both groups, this phase (chemotherapeutic or radiotherapy and antibiotic treatment before the infusion phase) is performed in the hospital.

The patients whose post-transplantation was performed at home presented a mean of 22.4 ± 2.6 days of the process, with a standard of 16.4 ± 2.08 post-transplant days. The patients whose post-transplantation was performed in the hospital presented a mean of 21.21 ± 4.18 days of the process, with a standard of 15.51 ± 3.96 days post-transplantation (days of the process *p* = 0.022; days post-transplantation *p* = 0.002). A significantly higher percentage of patients receiving both parenteral nutrition and packed red blood cells during the post-transplant phase was observed in the hospital setting. The rest of the therapeutic measures recorded did not present significant differences between the hospital/home contexts (Table 2).

Upon examining the incidence of adverse effects during the post-transplant phase, a noteworthy rise in neutropenic fever, mucositis, and positive blood cultures was evident among patients undergoing transplantation in the hospital (Table 3). Conversely, patients recovering at home exhibited a significantly more pronounced weight loss during the process.

Of the hospital patients with mucositis (18 persons), 10% reached grade III, while 66% of the home patients with mucositis (3 persons) reached grade III. Of the hospital patients who suffered vomiting (27 persons), 3.7% reached grade III, while none of the home patients with vomiting (11 persons) reached grade III. Of the hospital patients with diarrhea (32 persons), 3.1% reached grade III, while none of the home patients with diarrhea (17 persons) reached grade III.

Concerning the pathogens identified in blood cultures, up to nine were isolated in hospital patients, compared to two isolated in home patients. Multi-resistant pathogens were absent in-home patients, while 12.5% of hospital patients had such pathogens in their blood cultures.

Upon analyzing complications related to the catheter at the local level, it was noted that 25% of hospital patients experienced erythema, a condition observed in only 5% of home patients (*p* = 0.056). Regarding axillary, rectal, nasal, pharyngeal, and fecal colonization, up to eleven pathogens are detected in hospital patients, compared to five in-home patients. A total of 15% of the post-home transplant patients required readmission of more than 72 h to assess their evolution in the transplant process.

When the risk ratio is analyzed, it is identified that people undergoing HCT whose follow-up was conducted in a hospital setting are more likely to have certain complications than those with home follow-ups, such as neutropenic fever (OR 8.556), mucositis (OR 4.636), and presence of pathogens in blood cultures (OR 6.652).

## 4. Discussion

This study aimed to assess whether there are significant differences in post-transplant complications between HCT following the home or hospital modality, finding substantial differences in some post-transplant adverse effects.

We started from an initial sample of 20 post-transplant patients (total transplants performed in the period) in a home setting to whom 40 controls transplanted in the hospital were assigned. The baseline conditions of the sample did not present differences except for the assessment of the Karnofsky scale (with higher scores in the group of at-home patients). This scale shows the general state of health and the level of autonomy in the daily life of patients [40,41]. In different studies, it has been related to the appearance of further complications [42,43]. The patient’s level of autonomy is crucial for selecting patients in the HCT program at home since they must present an excellent general condition as an inclusion criterion. 

Regarding the days of admission, the whole process of patients at home reached an average of 22.4 ± 2.6 days, with an average of 16.4 ± 2.08 post-transplant days, compared to 21.21 ± 4.18 days of the process, with an average of 15.51 ± 3.96 days post-transplant in the hospital controls. In Grizzo’s study, the outpatient-based group had a markedly lower mean number of process days than hospitalized patients (22 vs. 47; *p* < 0.001) [44]. The difference in our study may be due to the selection of the controls (similar characteristics), which means there are no high differences in the baseline.

The pre-transplant antibiotic protocol is identical in both groups (all patients included in the program received pre-transplant prophylactic antibiotic therapy consisting of two antibiotics: a quinolone, levofloxacin, combined with a broad-spectrum antibiotic, ceftriaxone, or piperacillin/tazobactam, based on the experience of other groups [33,45]). However, there is a higher post-transplant antibiotic usage in the hospital group.

One of the most significant post-transplant complications is neutropenic fever. In the results obtained in this study, it can be observed that the incidence of neutropenic fever and the average duration of this fever are lower in the home transplant modality. This result is relevant, considering that neutropenic periods are significantly longer in patients studied at home. The results of previous studies show that antibiotic prophylaxis at home with quinolones associated with broad-spectrum antibiotics in home transplants was highly satisfactory compared to those performed in the hospital [33,46,47].

Malnutrition is a risk factor for adverse effects in patients undergoing HCT that directly and indirectly contributes to increased mortality and morbidity [48]. The results of our study show that hospitalized patients lost less weight than those who remained at home during the post-transplant period, despite not presenting significant differences in terms of the presence and duration of episodes of diarrhea, nausea, and vomiting, and even though episodes of mucositis occurred in a higher percentage in a hospital setting. These results make us consider that the lower hospital weight loss may be because hospitalized patients are administered continuous fluid therapy during admission, contributing to better hydration. To this must also be added the lack of freedom of movement by having to remain, throughout the admission, inside an isolation room without performing any exercise, which facilitates the appearance of edema. Likewise, it is observed that the prescription of parenteral nutrition (PN) is associated with episodes of mucositis in hospitalized patients, similar to what was seen in the work of Gonzalez-Barrera et al. [49]. Although the patients at home underwent greater weight loss, they did not report difficulties ingesting solid and liquid foods, which would have raised the need for NP in said group. These data lead us to consider that the hospitalized patient may be overmedicated and receive a greater extra caloric or fluid intake reflected in less weight loss, but not necessarily a better nutritional status. Studies such as that of Murray [50] and Gonzalez-Barrera [51] indicate that, whenever possible, and if there are no severe gastrointestinal problems, intravenous fluids and oral diet should be considered in preference to PN. At first, we thought that home patients having greater freedom to select the foods and choose how to cook them to their liking would mean that their food intake would be more significant and would influence less weight loss, but the results show the opposite. This study did not collect aspects such as caloric intake and other parameters to assess nutritional status beyond weight loss. 

On the other hand, in the case of hospitalized patients, as mentioned, there may be an excessive use of PN and IV hydration, which may increase the length of hospitalization and the possibility of developing complications related to venous access and its management. Regarding infectious complications, we have found references demonstrating that CVC-associated infections impact the delay in administering some treatments, prolong hospitalizations, and increase care costs and the mortality rate [52,53,54]. Although the results of our study do not show significant differences in complications and infections related to the catheter between the two modalities, a higher percentage of local complications was observed in patients who remained hospitalized. This could be attributed to the reduced catheter manipulation and the educational measures provided to family members and patients in the home setting [39].

Despite not detecting differences in catheter-related infections, we did find a significant difference in the percentage of patients with positive blood cultures in the hospital setting, where more post-transplant systemic diseases are collected. Likewise, the number of pathogens isolated in hospitalized patients’ blood cultures is more remarkable than in those isolated at home, and no multidrug-resistant pathogens were found in this context. Considering colonization, no significant differences were found in any anatomical location. However, again, the number of pathogens isolated in cultures is more important in the hospital than in the home context. Several studies suggest that outpatient care is safe and feasible during or after high-dose chemotherapy and HCT [24,34]. 

HCT centers have developed programs to provide more outpatient care under three basic models: an early discharge model, a late admission model, and a comprehensive or total outpatient model [55]. The implementation of these services has meant maintaining the quality of care with a reduction in costs [44], which has even led to improvements in the economic, social, and quality of life implications for patients in different studies, even when they return to work after treatment, which is considered a marker of functional recovery [56]. This study has shown that we must continue to advance our knowledge about caring for complex patients in home settings. As in other studies [50], verifying the non-inferiority of results and the safety for the patient when cared for at home opens alternative possibilities to hospital institutionalization as a care model; verification requires a proper patient selection process. However, many factors, such as facility experience, proximity to home/accommodation, reliable and available caregiver, and the patient’s treatment cost, determine this choice [33,54].

This study has the limitation of being a retrospective study in which a review of medical records was carried out. The exclusive review of medical records may need to be revised to obtain all the information, given that we depend on the evolutions having transcribed all the treatment, analytical results, and microbiological tests. Thus, during data collection, the possibility of recording biases was observed. For example, the ECOG variable was projected to define the patient’s baseline situation. However, it was not collected in all hospitalized patients, so it had to be left out of the analysis. Although an attempt was made to reduce this bias by training the researcher in charge of data collection and subsequent auditing of said collection, indeed, unregistered data could not be collected.

As future lines of research, the assessment of the quality of life aspects of both the patient and the family in both areas is proposed, as well as the establishment of strategies to improve care (patient and family training, increased nursing control) that allow for optimizing attention in the transplant processes and consequently improve said quality of life.

## 5. Conclusions

Regarding the differences in post-transplant adverse effects, we can recommend a home transplant as a safe therapeutic option that presents non-inferiority conditions compared to a hospital transplant. In our study, home patients show more significant weight loss than hospitalized patients, while in-hospital transplants increase the risk of episodes of neutropenic fever, mucositis, and positive blood cultures. 

## Figures and Tables

**Table 1:** Baseline conditions of patients undergoing transplantation according to home or hospital post-transplant setting.

Variable	Hospital *n* = 40	Domiciliary *n* = 20	*p*
Age	56.4 ± 11.21	56.25 ± 10.14	0.736
Male	60%	60%	1.000
Female	40%	40%	
Comorbidities	37.5%	45%	0.576
Diabetes	10%	15%	0.429
HBP	15%	30%	0.152
Dyslipidemia	15%	10%	0.461
KARNOFSKY	87.74 ± 7.62	91.76 ± 3.9	0.050
SORROR	1.95 ± 1.45	2.05 ± 1.39	0.808
Myeloma	75%	75%	1.000
Lymphoma	25%	25%	
CR	35.9%	50%	0.297
PR	64.1%	50%	

^1^HBP: High blood pressure; CR: complete remission; PR: partial remission.

**Table 2:** Hospital post-transplant therapy vs. domiciliary post-transplant therapy.

Variable	Hospital *n* = 40	Domiciliary *n* = 20	*p*
PN	19.4%	0%	0.036
Days having PN	6.75 [±3.32]	-	-
Post-transplant antibiotics	90%	80%	0.246
Number ATB	2.07 [±1.47]	1.75 [±1.48]	0.449
Days ATB	8.56 [±4.29]	7.29 [±1.93]	0.440
Antiviral	7.5%	0.%	0.289
Antifungal	2.5%	10.5%	0.240
Platelet transfusion (YES/NO)	100%	100%	-
Number of pool platelets	3.5 [±2.26]	4 [±1.65]	0.107
Transfusion CRBC (YES/NO)	72.5%	31.6%	0.003
Number of RBCC received	1.8 [±1.74]	0.42 [±0.76]	0.001

PN: parenteral nutrition; ATB: antibiotic; CRBC: concentrated red blood cells.

**Table 3:** Adverse effects post-hospital transplant vs. domiciliary conditions.

Variable	Hospital *n* = 40	Domiciliary *n* = 20	*p*
Neutropenic fever	87.5%	45%	0.000
Days with neutropenic fever	3.28 ± 2.9	0.89 ± 1.6	0.000
Days with neutropenia	9.08 ± 1.77	11.5 ± 2.5	0.000
Mucositis	45%	15%	0.022
Days with mucositis	4.21 ± 1.88	3.5 ± 0.7	0.670
Catheter infection	10%	5%	0.455
Catheter complications	42.5%	25%	0.149
Early catheter removal	30%	15%	0.206
Vomiting	69.2%	50%	0.123
Days vomiting	3.46 ± 2.5	2.45 ± 1.36	0.425
Diarrhea	80%	85%	0.464
Days with diarrhea	4.03 ± 2.05	5 ± 2.53	0.171
Fasting	12.5%	15.8%	0.510
Days fasting	2.6 ± 1.8	1.33 ± 0.57	0.337
Weight loss	3.41 ± 2.68	4.42 ± 1.71	0.018
Pathogen in blood cultures	42.5%	10%	0.011
Colonization	40%	35%	0.707
Axillary colonization	10%	15%	0.429
Rectal colonization	20%	15%	0.464
Pharyngeal colonization	15%	5%	0.247
Nasal colonization	12.5%	5%	0.340
Pathogen in stool	10%	15%	0.429
Respiratory complications	12.5%	0%	0.120
Circulatory complications	7.5%	0%	0.289
Renal complications	2.5%	0%	0.667
Electrolyte complications	7.5%	15%	0.314

## Data Availability

Data are available upon request from the corresponding author.
